# Chronic morbidity and reported disability among older persons from the India Human Development Survey

**DOI:** 10.1186/s12877-018-0979-9

**Published:** 2018-12-07

**Authors:** Mukesh C. Parmar, Nandita Saikia

**Affiliations:** 1 Centre for the Study of Regional Development, School of Social Sciences, Jawaharlal Nehru University, New Delhi, India; 2 International Institute for Applied Systems Analysis, Laxenburg, Austria

**Keywords:** Activities of daily living, ADL, Ageing, Disability, Morbidity, India Human Development Survey, IHDS, India, Katz index, Katz score

## Abstract

**Background:**

The burden of disability and chronic morbidity among the elderly has been increasing substantially in India in recent years. Yet, the use of nationally representative data to investigate the relationship between chronic morbidity and reported disability in the country has been minimal. The objective of this study is twofold: i) to quantify the association between chronic morbidities and overall disabilities in the activities of daily living (ADLs) among elderly people in India, and ii) to understand how various chronic morbidities influence individual ADLs, specifically, walking, toileting and dressing.

**Methods:**

We used data from the India Human Development Survey-II (IHDS-II) as a basis for this study. We computed the Katz Index of independence in ADL to examine the burden of disability among the elderly. Ordered logistic regression was carried out to examine the effect of chronic morbidities on: i) the disability index (where 0 = no disability; 1 = disability in 1 or 2 ADLs; and 2 = disability in 3 ADLs), and ii) disabilities in three ADLs in the population over-60 years of age in India.

**Results:**

The percentage of people scoring lower Katz index (indicating severe and mild disability) in at least one of the three ADLs is very high in India (17.91% for males and 26.21% for females). Irrespective of the type of ADL, the Katz score is lower in elderly females than in elderly males. Elderly people who are illiterate and belong to the poorest wealth quintile report lower Katz scores in ADL. Both bivariate and multivariate analyses confirm that all three types of chronic morbidities are positively and significantly associated with a disability condition in the ADLs. Yet, the effects of morbidities vary greatly according to the type of disability. For instance, while diabetes affect walking (OR: 2.56; 95% CI: 2.29–2.86), and toileting (OR: 2.63; 95% CI: 2.26–3.07), high blood pressure mainly affects walking (OR: 2.29, 95% CI: 2.09–2.5) and dressing disabilities (OR: 2.13, 95% CI: 1.84–2.46).

**Conclusions:**

Chronic morbidity is a decisive factor in old age disability**.** It is crucial to reduce chronic morbidity in a timely way to minimise the enormous associated burden of disability.

## Background

According to the 2011 census, India is home to 27 million people with severe disabilities [1]. Around 5% of the elderly population in the country are affected by some kind of disability [2], and the burden is predicted to increase substantially due to rising life expectancy and associated population aging. Despite this, studies addressing disability dynamics in India are limited.

Research in several other countries has shown that there is a strong association between chronic morbidities and disabilities [3, 4]. As severe morbidity disrupts normal daily activities, it reduces quality of life [5–8]. Some studies have found that arthritis [4, 9, 10], cardiovascular disease, lung disorders, vision syndrome disabilities, and diabetes [9, 11] are common causes of physical disability. Depression and other major chronic diseases [12–14] can lead to mental disability. The interaction of various chronic diseases with physical disability [15] and the combined effects of two or more diseases [16] have also been conceptualised.

A literature survey on disability shows that the studies addressing disability in India mainly revolve around three principal issues. These are: i) the measurement of disability data in census and other surveys [17–19]; ii) differentials, determinants, and the impact of disability in general [2, 17, 20–23]; and iii) the pattern of disability regarding the health of older adults [19, 24–28]. In the first type of study, there is considerable debate on the actual prevalence of disability in India. For instance, the national sample survey (NSSO) and the national census are the two main data sources used in the measurement of disability in the country, but these differ in sample design, definition, and description of disability type [17–19]. In these studies, disability is measured in terms of disability prevalence, the disability index, and the age-standardized disability rate [1, 2, 29].

The second type of study focuses on the association between socioeconomic variables and disability. Females are found to be at higher risk of disability in old age than men [1, 2, 9, 30], but this gender gap has been decreasing over time [31]. Researchers have pointed out that age [4, 32] and education [6, 33, 34] influence the disability status of the elderly.

The third type of study also deals with the interrelation between morbidity and disability [29, 35]. These studies from India show that diseases like arthritis, hypertension, diabetes, and other physiological disorders are all linked to disability in old age [35]. Several studies have addressed the effect of morbidities on the ADLs of elderly people (60+), such as i) the effect of diabetes and other co-morbidities, like heart disease and hypertension on walking disability and other physical activities; ii) the effect of cataracts on vision syndrome disability [36]; and iii) mental disorders that cause hearing and speech impairments [37]. The states of Chandigarh and Haryana (2006) have revealed similar results, highlighting the strong association between morbidity and disability [29].

Previous studies conducted in India, however have two major limitations. First, they are based on primary sample survey, which restricts their study area to small geographical pockets of India and thus provide only a small sample size. Findings from these studies can therefore not be generalized to understand the nature and extent of the relationship between morbidities and disabilities for a large and heterogeneous country like India. Second, as all these studies have focused on the association between a specific type of disability and a set of morbidities, they have failed to provide a broad picture of the relative importance of morbidities for various types of disabilities among the elderly using the same study design. In the present study, we first examine the role of three important old-age morbidities on the overall disability status of elderly people in India using a recently available nationally representative survey. We then examine how each type of morbidity affects each type of disability in ADLs among the elderly in the country. The first part of the analysis helps to understand the relative importance of morbidities for the overall disability condition of the elderly, while the second part reveals the relative effect of each morbidity on each type of disability.

## Methods

### Data description

We used data from the second round of the India Human Development Survey (IHDS-II) [38], which was conducted in 2011–2012 and contains nationally representative longitudinal panel data. The survey covered about 42,152 households and 53,582 individuals across India. It was coordinated by the University of Maryland and the National Council of Applied Economic Research (NCAER), New Delhi. The survey covered a wide range of topics like health, employment, socioeconomic aspects, agriculture, economy, and education. A stratified random sampling technique was used for the survey. In the second round of the IHDS survey, information on morbidity and reported disability was recorded in the form of fifteen types of chronic morbidities and disabilities in seven types of ADL.

### Measurements

#### Dependent variables

In this study, we define disability as “difficulty or inability” in the performance of ADLs by household members aged 60 and above.

In the present study, we considered disability in three ADLs for older persons in India, namely, walking, toileting and dressing. The exact question asked in the survey was: “Now, I am going to ask you about any physical difficulty that people above the age of 60 in this household may have. Does anyone in the household have a problem?” If the response is “yes,” the next question is: “Can (name of the affected person) still do it with some trouble or is he/she unable to do it?” with options for three types of ADL: “i) walking 1 km; ii) going to the toilet without help; iii) dressing without help”. All these options have three responses, namely, “No difficulty”, “Can do with difficulty” and “Unable to do it” . The respondents to the above questions were women between the ages of 15 and 49 years matching the study criteria. We computed the Katz index of independence in ADL, referred to as the ‘Kz score’, to assign coding to these categories. Hence, a Kz score = 0 signifies “Unable to do it”, a Kz score = 1 signifies “Can do with difficulty”, and a Kz score = 2 signifies “No difficulty”. The Katz Index of Independence in ADL, also called “Katz ADL” [39] is the standardised index for measuring the degree of functionality in ADL.

Similarly, Katz ADL is used to create a disability index to comprehend the effect of multiple disabilities in a sampled population. Here, we first categorized all types of disabilities in binary form, namely, “0” (combining “can do it with difficulty” and “unable to do it”) and “1” (no difficulty or functional independence of a person). The scores for all three types of disability were then added to create the ‘Kz score’. Finally, this combined score was split into three groups, namely, “Kz score = 3” (no difficulty in any ADL), “Kz score=1 or 2” (mild or severe disability in one or two ADLs), and “Kz score = 0” (mild or severe disability in all three ADLs). Thus, an elderly person with a Kz score of less than 3 was suffering from multiple disabilities at the time of the interview.

Secondly, we took each of the ADLs (walking, toileting and dressing) as dependent variables (categorized into “no difficulty,” “can do with difficulty” and “unable to do it”) to examine the role of morbidities according to type of disability.

#### Exposure variables

The main exposure variable in our analysis was the presence of any three types of chronic morbidities, specifically high blood pressure (high BP), heart disease and diabetes. The exact question was: “Has a doctor ever diagnosed any member in the household as having – high BP/heart disease/diabetes?”

Following the available literature, we included a set of demographic and socioeconomic variables in the regression model. These are: age (60–69, 70–79, 80–89); sex (male or female); place of residence (rural or urban); marital status (currently married, never married and other); religion (Hindu, Muslim and other), caste (scheduled caste, scheduled tribe and other), wealth index quintiles (Q1, Q2, Q3, Q4, Q5); and level of education (no education, primary, secondary or higher).

To clarify the terminology of the variables above, we define caste as a ‘social stratification’ in the Hindu religion where there are four *Varnas* or tiers: *Brahmin* (priest and teacher), *Kshatriya* (ruler and warrior), *Vaishya* (trader), and *Shudra* (servant), which are further divided into smaller social groups. The *dailts* (untouchables) and *adivasis* (tribals) are considered to be outside of the caste system and are therefore seen as the lowest in the social hierarchy. They are referred to as scheduled caste (SC) and scheduled tribe (ST) respectively [40]. The constitution of India urges positive measures for the people belonging to the SC and ST community in order to uplift their socioeconomic status. The variable wealth quintile index, on the other hand, is generated by dividing the total household income given in the dataset of the IHDS (from agriculture, livestock, salaries, wages, non-farm business, property and other assets, pensions, scholarships and government benefits) into five equal parts namely, Q1, Q2, Q3, Q4 and Q5 in ascending order, thus representing the economic hierarchy.

### Statistical analysis

First, we cross-tabulated the dependent and the independent variables to understand the distribution of the disability status across demographic and socioeconomic characteristics. Ordered logistic regression was carried out to examine the relationship between three types of morbidity and disability indices, based on three ADLs. In addition, ordered logistic regression was carried out to examine the relationship between three types of morbidity and each of the three types of ADLs listed above. To test the proportional odds assumption for ordinal logistic regression, the Brant Test was conducted. The proportional odds assumption, or the parallel odds assumption, states that each pair of response variables has the same relationship from the lowest through to the highest category, as described in the variable. A post-estimation test was also done to check the multi-collinearity between high BP, heart disease and diabetes. All the analyses were conducted on a population older than 60 years, using the STATA statistical software package version 14.

## Results

### Prevalence of disability in three ADLs by gender

Table 1 presents Katz’s score in ADL (expressed in %) among the population aged 60 and above in India, 2011–2012. This table reveals many important findings about the status of disability among the elderly in India.

**Table 1:** Katz (Kz) score in ADL (expressed in %) among population aged 60 and above in India, 2011–2012

ADL	Male (*N* = 10,523)			Female (*N* = 11,402)		
	Severe(Kz score = 0)%	Mild(Kz score = 1) %	Non-disabled(Kz score = 2)%	Severe(Kz score = 0)%	Mild(Kz score = 1) %	Non-disabled(Kz score = 2)%
Walking	6.02	11.67	82.31	9.48	16.32	74.19
Toileting	2.34	4.79	92.87	3.45	7.07	89.47
Dressing	1.98	2.85	95.17	2.98	4.86	92.16
At least one of above	6.66	11.27	82.07	10.51	15.7	73.79

Kz score = 0: Unable to perform ADL; Kz score = 1: Can do with difficulty ADL; Kz score = 2: fully functional ADL

First, the percentage of people over 60 obtaining lower Katz scores (indicating either mild or severe disability) in any of the three ADLs is very high (17.93% for men and 26.21% for women). For both genders, the Katz score of mild disability in ADLs is much higher than that of severe disability. Second, irrespective of the type of ADL and the severity of the corresponding disability, the Katz scores for severe disability in ADLs are higher in elderly females than in elderly males. Finally, the Katz score of severe disability in walking for both genders is the highest (for Kz score = 0, 6.02% for men vs 9.48% for women; for Kz score = 1, 11.67% for men vs 16.32% for women). Disability in walking was followed by disability in toileting (for Kz score = 0, 2.34% for men vs 3.45% for women, and for Kz score = 1, 4.79% for men vs 7.07% for women).

### Disability status by socioeconomic characteristics and type of morbidity

Table 2 presents the disability prevalence expressed as Katz’s scores in ADL (in %) in the Indian population aged 60 and above by demographic and socioeconomic characteristics.

**Table 2:** Disability prevalence expressed as Katz score in ADL (in %) in population aged 60 and above by demographic and socioeconomic characteristics, India, 2011–2012

Characteristics	N	Kz score = 0	Kz score = 1 or 2	Kz score = 3
Chronic morbidity				
High BP				
No	19,421	5.16	15.08	79.77
Yes	2504	10.58	26.8	62.62
Heart diseases				
No	21,318	5.68	16.13	78.19
Yes	607	9.03	26.46	64.51
Diabetes				
No	20,467	5.45	15.77	78.78
Yes	1458	10.34	25.47	64.18
Sex				
Male	10,636	4.43	13.5	82.07
Female	11,289	7.04	19.16	73.79
Age				
60–64	7389	2.40	10.62	86.98
65–69	5802	3.71	14.55	81.74
70–74	3916	6.20	19.35	74.44
75–79	2254	8.21	23	68.8
80+	2563	17.37	27.11	55.51
Place of residence				
Rural	15,442	5.71	16.98	77.31
Urban	6483	5.92	15.09	79
Marital status				
Currently married	13,213	3.77	13.43	82.81
Never married	222	4.71	18.53	76.76
Other	8490	8.93	21.02	70.05
Religion				
Hindu	18,410	5.71	16.52	77.77
Muslim	2133	6.82	14.69	78.49
Other	1382	5.02	17.77	77.21
Caste				
SC	4238	5.65	16.17	78.18
ST	1400	4.11	11.36	84.53
Other	16,227	5.95	16.93	77.12
Wealth				
Q1	6301	6.17	18.66	75.17
Q2	4088	6.68	14.91	78.41
Q3	3755	5.58	16.76	77.66
Q4	3977	5.06	14.71	80.22
Q5	3804	5.09	15.77	79.14
Education				
No education	12,921	6.59	18	75.41
Primary	3829	5.31	16.14	78.55
Secondary	3683	4.56	13.32	82.12
Higher	1449	2.95	10.66	86.39
Total	21,926	5.45	16.88	77.67

Disability prevalence is measured by Katz score (Kz score) which is constructed using the Katz Index of Independence in ADL with three categories, specifically: Disability in three ADLs: Kz score = 0; Disability in 1 or 2 ADLs: Kz score = 1 or 2; Fully functional ADL: Kz score = 3. Functional independence increases with higher Kz score

The most striking finding of Table 2 is that the lowest Katz score in ADLs (Kz = 0), that is, disability in all three ADLs, is higher among people diagnosed with chronic morbidities. For instance, the prevalence rate of disability in elderly people with diabetes and high BP is double (for Kz score < 3) than for elderly persons without these morbidities (diabetes: 10.34% vs. 5.45%; high BP: 10.58% vs. 5.16%).

In general, the percentage of women with Katz scores of higher disability in multiple ADLs is higher than that of men. Moreover, as age increases, the Katz score of disability in ADL also rises. There is a marginal difference in disability prevalence by type of residence and religion. The results also indicate that widows or divorcees (included in the “other” category in marital status) are at higher risk of suffering from disability than their married counterparts. Katz scores of higher disability in three ADLs (Kz score = 0) is more prevalent among older Muslims than among elderly people from other religions (Hindu: 5.71%; Muslims: 6.82% and other: 5.02%). The disability rate is lowest among older people belonging to the ST (4.11%). No clear downward gradient between the disability rate and wealth index is observed, although the lowest Katz score in ADL (Kz score = 0) is observed among those in the lowest wealth quintile. Disability rates are reduced substantially from the uneducated to the higher-educated group (for Kz score = 0, uneducated: 6.59%, higher educated 2.95%).

### Type of disability by chronic morbidities and socioeconomic characteristics

Table 3 presents Katz scores in each ADL (expressed in %) in the population aged 60 and above by socioeconomic characteristics. The Katz score of highest disability in ADLs (Kz score < 2) was found to be the highest among the elderly suffering from high BP. Walking-related disability was also prominent among the elderly with major morbidities like cardiovascular disease and diabetes. Among high BP patients, mild disability (Kz score = 1) in toileting and dressing is 11.37 and 7.21%, respectively. About 24.55% of diabetes patients have mild walking-related disabilities (Kz score = 1).

**Table 3:** Katz score in each ADL (expressed in %) in population aged 60 and above by socioeconomic characteristics, India, 2011–2012

Characteristics		Walking			Toileting			Walking		
	N	Severe(Kz score = 0)%	Mild(Kz score = 1) %	Non-disabled(Kz score = 2)	Severe(Kz score = 0)%	Mild(Kz score = 1) %	Non-disabled(Kz score = 2)	Severe(Kz score = 0)%	Mild(Kz score = 1) %	Non-disabled(Kz score = 2)
Chronic morbidity										
High BP										
No	19,191	7.20	12.70	80.1	2.77	5.27	91.96	2.30	3.45	94.25
Yes	2734	12.50	24.64	62.86	4.01	11.37	84.62	4.00	7.21	88.79
Heart diseases										
No	21,253	7.67	13.81	78.52	2.89	5.85	91.26	2.48	3.81	93.71
Yes	672	12.25	23.11	64.64	3.71	10.07	86.22	3.21	6.28	90.51
Diabetes										
No	20,334	7.57	13.32	79.11	2.82	5.60	91.58	2.42	3.65	93.93
Yes	1591	11.03	24.55	84.71	4.15	11.14	84.71	3.63	7.08	89.29
Sex										
Male	10,636	6.02	11.67	82.32	2.34	4.79	92.88	1.98	2.85	95.17
Female	11,289	9.48	16.32	74.19	3.45	7.07	89.47	2.98	4.86	92.16
Age										
60–64	7389	3.75	8.92	87.33	1.25	2.82	95.93	1.02	1.82	97.16
65–69	5802	5.32	12.74	81.94	2.01	4.28	93.71	1.96	2.29	95.75
70–74	3916	8.83	16.48	74.7	2.62	6.77	90.6	2.19	4.53	93.28
75–79	2254	11.42	19.45	69.13	4.44	8.28	87.28	3.86	5.04	91.10
80+	2563	20.37	23.47	56.15	8.83	15.59	75.58	7.25	11.41	81.34
Place of residence										
Rural	15,442	7.88	14.42	77.7	2.98	6.12	90.91	2.51	3.82	93.67
Urban	6483	7.62	13.21	79.17	2.75	5.60	91.64	2.47	4.01	93.52
Marital status										
Currently married	13,213	5.34	11.55	83.11	1.99	4.35	93.66	1.71	2.55	95.75
Never married	222	6.67	16.33	77	2.52	5.46	92.02	0.32	4.62	95.06
Other	8490	11.66	17.91	70.43	4.36	8.49	87.15	3.79	5.94	90.28
Religion										
Hindu	18,410	7.68	14.22	78.1	2.83	5.99	91.18	2.39	3.93	93.68
Muslim	2133	8.00	13.15	78.85	3.69	6.38	89.93	3.23	4.24	92.53
Other	1382	9.12	13.42	77.46	2.85	4.96	92.18	2.80	2.62	94.59
Caste										
SC	4238	8.74	12.83	78.44	3.20	5.65	91.16	2.57	3.50	93.92
ST	1400	5.04	10.02	84.95	2.96	3.70	93.34	2.90	1.95	95.15
Other	16,227	7.79	14.75	77.46	2.84	6.23	90.93	2.44	4.14	93.42
Wealth										
Q1	6301	7.79	16.46	75.75	3.09	7.02	89.9	2.75	4.20	93.04
Q2	4088	8.76	12.56	78.68	3.25	6.26	90.49	2.53	4.71	92.76
Q3	3755	8.20	13.92	77.88	2.92	5.43	91.65	2.50	3.87	93.63
Q4	3977	7.49	11.99	80.52	2.54	4.79	92.66	2.16	3.46	94.38
Q5	3804	6.71	14.02	79.27	2.63	5.66	91.71	2.38	2.90	94.71
Education										
No education	12,921	8.86	15.25	75.89	3.37	6.51	90.13	2.93	4.50	92.57
Primary	3829	7.17	14.19	78.65	2.52	6.74	90.74	2.08	3.55	94.37
Secondary	3683	6.11	11.64	82.24	2.39	4.39	93.23	1.98	2.74	95.28
Higher	1449	4.31	9.14	86.55	1.31	3.07	95.62	1.13	2.15	96.72

Kz score = 0: Unable to perform ADL; Kz score = 1: Can do with difficulty ADL; Kz score = 2: fully functional ADL

Consistent with Table 2, females are found to bear an unequal burden of disability, most noticeably in walking (for Kz score = 1, walking = 16.32%, toileting = 7.07%, and dressing = 4.86%). Most of the disabled population come from the 60–64 age group, but the highest prevalence of disability is observed in the over-80 age group. The ranking of disability prevalence for mild and severe disability for the over-80 population is as follows: walking (Kz score = 1: 23%, Kz score = 0: 20%), toileting (Kz score = 1: 15%, Kz score = 0: 8%) and dressing (Kz score = 1: 11%, Kz score = 0: 7%). This ranking remained almost the same in all other socioeconomic subgroups. Mild disability is more prevalent than severe disability across all categories.

Another distinctive feature of Table 3 is that most of the elderly with mild or severe (Kz score < 2) disabilities belong to the poor wealth quintile and are illiterate. Progressing through the higher categories of each distinctive group of wealth quintile and education, one sees the situation improving a little. However, the highest wealth quintile is seen to report a high mild disability (Kz score = 1) prevalence for walking.

Disabilities of toileting and dressing-related ADLs are more prevalent among the Muslim than the Hindu population. People belonging to the STs have the least prevalence of disability, compared to those from the SC and other castes. Clearly visible for SC, ST and other castes, for example, is the difference in the prevalence of severe disability (Kz score = 0) in walking (SC: 8.74%, ST: 5% and other: 7.79%) and mild disability (Kz score = 1) for dressing (SC: 3.5%, ST: 1.95% and other: 4.14%).

### Role of morbidity on disability: Results of regression analysis

Table 4 shows the results of the ordered logistic regression revealing the role of chronic morbidity in disability in ADLs.

**Table 4:** Ordered logistic regression analysis of disability prevalence among population aged 60+ in India, 2011–2012

Background variable	Odds ratio	*p*-value	95% Confidence interval	
			Lower	Upper
Chronic morbidity				
High BP (No^a^, Yes)	1.94	*p* < 0.0001	1.76	2.13
Heart diseases (No^a^, Yes)	1.80	*p* < 0.0001	1.52	2.13
Diabetes (No^a^, Yes)	2.03	*p* < 0.0001	1.80	2.29
Sex				
Male^a^				
Female	1.35	*p* < 0.0001	1.25	1.46
Age	1.07	*p* < 0.0001	1.07	1.08
Education				
Illiterate^a^				
Primary	0.99	0.8060	0.90	1.09
Secondary	0.85	0.0050	0.77	0.95
Higher	0.68	*p* < 0.0001	0.57	0.81
Wealth				
Poorest^a^				
Poor	0.80	*p* < 0.0001	0.72	0.88
Middle	0.85	0.0020	0.77	0.95
Rich	0.70	*p* < 0.0001	0.64	0.78
Richest	0.66	*p* < 0.0001	0.60	0.74
Religion				
Hindu^a^				
Muslim	1.01	0.8550	0.90	1.13
Other	1.00	0.9520	0.87	1.13
Caste				
SC^a^				
ST	0.83	0.0200	0.70	0.97
Other	1.05	0.3290	0.96	1.14
Residence				
Urban^a^				
Rural	0.89	0.0030	0.82	0.96
Marital status				
Currently married^a^				
Never married	1.08	0.6790	0.76	1.52
Other	1.27	*p* < 0.0001	1.17	1.37

Dependent variable: Disability index with three categories viz. No ADL, 1–2 ADL, > = 3 ADL

^a^Reference category

It is clear from Table 4 that almost all chronic morbidities have a statistically significant relationship with disability conditions in ADLs. Elderly people suffering from any type of chronic morbidity have at least a 1.8 times greater chance of having a disability condition, than those not suffering from any chronic morbidity. Elderly people suffering from diabetes (odds ratio (OR): 2.03, 95% confidence interval (CI): 1.80–2.29%) were more likely to report disability, compared to adults suffering from heart disease (OR: 1.8, 95% CI: 1.52–2.13) and high BP (OR: 1.94, 95% CI: 1.76–2.13). Overall, the proportion of elderly people with a higher level of education and income reported old age disability the least. Compared to currently married elderly persons, the divorced/separated elderly (counted in the ‘other’ category) report a higher incidence of disability (OR: 1.27, 95% CI: 1.17–1.37).

Table 5 presents the ordered logistic regression results of three different types of disability. All the models are adjusted for gender, age, education, wealth quintile, caste, religion and place of residence. It confirms that all the listed morbidities have a significant effect on a specific type of ADL. Diabetes appeared as the biggest threat to walking (OR: 2.56; 95% CI: 2.29–2.86), toileting (OR: 2.63; 95% CI: 2.26–3.07) and dressing- (OR: 2.5; 95% CI: 2.09–2.99). High BP mainly affects walking (OR: 2.29; 95% CI: 2.09–2.5) and dressing disabilities (OR: 2.13; 95% CI: 1.84–2.46).

**Table 5:** Ordered logistic regression analysis of specific disability prevalence among population aged 60+ in India, 2011–2012

	Dependent variable	Independent variable	Odds ratio	*p*-value	95% Confidence interval	
					Lower	Upper
Model 1	Walking	High BP	2.29	*p* < 0.0001	2.09	2.50
		Heart diseases	2.31	*p* < 0.0001	1.96	2.71
		Diabetes	2.56	*p* < 0.0001	2.29	2.86
Model 2	Toileting	High BP	2.10	*p* < 0.0001	1.85	2.39
		Heart diseases	2.06	*p* < 0.0001	1.64	2.58
		Diabetes	2.63	*p* < 0.0001	2.26	3.07
Model 3	Dressing	High BP	2.13	*p* < 0.0001	1.84	2.46
		Heart diseases	1.99	*p* < 0.0001	1.54	2.59
		Diabetes	2.50	*p* < 0.0001	2.09	2.99

Each model in the above table is adjusted for sex, age, education, wealth, religion, caste, residence and marital status

Heart disease least affects dressing-related disability as compared to high BP and diabetes.

## Discussion

As a result of increased population aging, the importance of disability studies among the elderly in India has been increasing for decades. The aim of this study has been to examine the nature and magnitude of the association between chronic morbidities and disability among the elderly in the country, using recently available nationally representative data. Unlike previous studies focusing on a specific disability, we documented the association between a set of major morbidities and disability conditions across three ADLs.

One important finding of this study is that the burden of disability among the elderly is enormous in India. Our study shows that about 17.93% of elderly men and 26.21% of elderly women in the country experience either mild or severe disability in three ADLs needed for a better quality of life. This indicates that about 9 million elderly men and 14 million elderly women in India have at least one type of disability, according to IHDS data. The ADL-based disability estimates presented in the current study is far higher than the disability estimates in the Indian census [1].

Our study demonstrates that disability prevalence is higher among women, those not currently married, and the oldest of the elderly (above 80 years). Wealthy elderly people experience a lower prevalence of disability than the poor do. The odds of experiencing disability decreases as level of education increases. Contrary to previous findings [1], disability prevalence is lower among the ST population. This may be the result of under-reporting, as self-reported health or disability, being a subjective measure, may depend on the level of awareness about health or disability. This needs further detailed investigation.

In accordance with previous research, our study also confirms that there is a strong positive association between the existence of any morbidity and any disability in ADLs [3, 41, 42]. One of the major findings of our study is that diabetes causes the highest likelihood of any disability among the elderly, followed by high BP and heart disease. Another major finding of our study is that we were able to quantify the relative role of morbidities for each specific disability. For instance, according to our study, the likelihood of disability is always the highest among diabetes patients, whereas the disability rate is the lowest among elderly persons with heart disease. This may be due to mortality selection among heart patients. It is found that heart disease is the topmost cause of death in India, whereas diabetes is the seventh most common cause of death [42]. Previous studies show heart disease and diabetes as a major contributing factor in disability [43] but this study gives relative role of heart disease and diabetes in the disablement process. These results are helpful for both patients and healthcare providers in terms of taking preventive measures at the onset of morbidities.

The strength of our study is the fact that we use information from recently available population based survey data. This helps to give a clear picture about the level of disability prevalence and disparities at the national level. To our knowledge, this is the first systematic study to analyse the association between various morbidities and disability conditions among the elderly at the national level in the country. Thus, our study is an important contribution to the understanding of disability conditions in India, which is one of the fastest aging countries in the world.

There are certain limitations to the current study. First, the IHDS data provides information on the disability conditions of the elderly, as reported by women (aged 15–49) in households matching the study criteria. Disability information from proxy respondents may be a limitation of this study. The literature suggests that in some cases, self-reporting and proxy reporting were correlated, while in other cases there were discrepancies regarding the disability condition of the elderly [44]. Yet, the direction of reporting (either under-reporting or over-reporting) by proxy respondents is not very conclusive in the relevant literature [45]. We therefore minimized the bias caused by proxy reporting by constructing a disability index.

Second, due to the small sample size, we could not encompass the individual influence of some of the major diseases such as sexually transmitted diseases (STDs) and AIDS, polio, cancer, leprosy, etc., on disability. It would have been preferable for the sample size to be large enough to analyse each disease and ADL separately.

## Conclusions

This study reveals that the burden of disability among the elderly in India is enormous and there is a strong relation between chronic morbidity and disability in them. More specifically, diabetes is an important contributing factor to disability than heart diseases. This burden may increase in the near future if public-health policies are not formulated and executed in a timely fashion. The burden of unhealthy aging can be a serious threat to sustainable economic development in the country. To address the present condition, it is important to expand societal and institutional support to the disabled elderly. Healthcare, transport, and other community-level facilities should be conducive to helping disabled elderly persons live out their lives with dignity. Due to the changing family system and related values, the elderly, particularly the disabled elderly, are susceptible to various kinds of abuse. Government assistance to families with disabled elderly members is thus much needed.

At the same time, an environment to prepare adults for healthy aging is the message of the moment. As morbidity is a major risk factor influencing disability in the elderly, a social environment should be created for early detection and postponing the onset of morbidity as the later stages of life approaches, by focusing on a healthy lifestyle from the beginning of adulthood. To achieve this, all stakeholders including government, community health workers, and civil society, need to play an essential role.

## Abbreviations

ADL: Activities of daily living
BP: Blood pressure
IHDS: India Human Development Survey
NCAER: National Council of Applied Economic Research
OR: Odds ratio
SC: Scheduled caste
ST: Scheduled tribe
